# Supplementary material for: ProteinShader: illustrative rendering of macromolecules
Source: BMC Struct Biol. 2009 Mar 30;9:19. doi: 10.1186/1472-6807-9-19 (PMC2672931; doi:10.1186/1472-6807-9-19)
Supplement: Additional file 1 — ProteinShader program without source code. This compressed file contains the complete ProteinShader program including associated libraries, but no source code. A README.txt file gives an overview of the ProteinShader distribution, and the index.html file in the help subdirectory has directions on getting started with the program as well as a set of tutorials. [file 1472-6807-9-19-S1.zip › ProteinShader-beta-0_9_4-binary/help/api/org/proteinshader/gui/listeners/controlpanel/DecorationsPanelListenerFactory.html]

DecorationsPanelListenerFactory (ProteinShader API)


|  |  |  |
| --- | --- | --- |
| **Overview** \| **Package** \| **Class** \| **Use** \| **Tree** \| **Deprecated** \| **Index** \| **Help** | |  |
| **PREV CLASS**   **NEXT CLASS** | **FRAMES**    **NO FRAMES**     **All Classes** |  |
| SUMMARY: NESTED \| FIELD \| CONSTR \| METHOD | DETAIL: FIELD \| CONSTR \| METHOD |  |


---


## org.proteinshader.gui.listeners.controlpanel Class DecorationsPanelListenerFactory

```
java.lang.Object
  org.proteinshader.gui.listeners.controlpanel.DecorationsPanelListenerFactory
```

---

``` public class DecorationsPanelListenerFactory extends Object ```

Creates the listeners for the DecorationsPanel.

---

**Constructor Summary**

`DecorationsPanelListenerFactory(Mediator mediator,
DecorationsPanel panel)`
  
          Constructs a DecorationsPanelListenerFactory.


| **Method Summary** | |
| --- | --- |
| `ActionListener` | `createBendMenuActionListener()`             Creates an action listener for the 'Bend Texture:' menu. |
| `ActionListener` | `createExtraLinesCheckBoxActionListener()`             Creates and action listener for the "Extra Lines" check box. |
| `ActionListener` | `createHalftoningButtonActionListener()`             Creates an action listener for the 'Halftoning' radio button. |
| `ActionListener` | `createHalftoningMenuActionListener()`             Creates an action listener for the 'Halftoning Texture:' menu. |
| `ActionListener` | `createPatternsButtonActionListener()`             Creates an action listener for the 'Patterns' radio button. |
| `ActionListener` | `createPatternsMenuActionListener()`             Creates an action listener for the 'Patterns' texture menu. |
| `ActionListener` | `createPlainButtonActionListener()`             Creates an action listener for the 'Plain' radio button. |
| `ActionListener` | `createTextLabelsButtonActionListener()`             Creates an action listener for the 'Text Labels' radio button. |
| `ActionListener` | `createWireframeButtonActionListener()`             Creates an action listener for the 'Wireframe' radio button. |

**Methods inherited from class java.lang.Object**

`clone, equals, finalize, getClass, hashCode, notify, notifyAll, toString, wait, wait, wait`

**Constructor Detail**

### DecorationsPanelListenerFactory

```
public DecorationsPanelListenerFactory(Mediator mediator,
                                       DecorationsPanel panel)
```

:   Constructs a DecorationsPanelListenerFactory.

    **Parameters:**: `mediator` - the centralized Mediator object that listeners call on to accomplish their task.: `panel` - the DecorationsPanel that this class creates listeners for.


**Method Detail**

### createPlainButtonActionListener

```
public ActionListener createPlainButtonActionListener()
```

:   Creates an action listener for the 'Plain' radio button.

    :   **Returns:**: The action listener.

---


### createWireframeButtonActionListener

```
public ActionListener createWireframeButtonActionListener()
```

:   Creates an action listener for the 'Wireframe' radio button.

    :   **Returns:**: The action listener.

---


### createTextLabelsButtonActionListener

```
public ActionListener createTextLabelsButtonActionListener()
```

:   Creates an action listener for the 'Text Labels' radio button.

    :   **Returns:**: The action listener.

---


### createPatternsButtonActionListener

```
public ActionListener createPatternsButtonActionListener()
```

:   Creates an action listener for the 'Patterns' radio button.

    :   **Returns:**: The action listener.

---


### createPatternsMenuActionListener

```
public ActionListener createPatternsMenuActionListener()
```

:   Creates an action listener for the 'Patterns' texture menu.

    :   **Returns:**: The action listener.

---


### createHalftoningButtonActionListener

```
public ActionListener createHalftoningButtonActionListener()
```

:   Creates an action listener for the 'Halftoning' radio button.

    :   **Returns:**: The action listener.

---


### createHalftoningMenuActionListener

```
public ActionListener createHalftoningMenuActionListener()
```

:   Creates an action listener for the 'Halftoning Texture:' menu.

    :   **Returns:**: The action listener.

---


### createBendMenuActionListener

```
public ActionListener createBendMenuActionListener()
```

:   Creates an action listener for the 'Bend Texture:' menu.

    :   **Returns:**: The action listener.

---


### createExtraLinesCheckBoxActionListener

```
public ActionListener createExtraLinesCheckBoxActionListener()
```

:   Creates and action listener for the "Extra Lines" check box.

    :   **Returns:**: The action listener.


---


|  |  |  |
| --- | --- | --- |
| **Overview** \| **Package** \| **Class** \| **Use** \| **Tree** \| **Deprecated** \| **Index** \| **Help** | |  |
| **PREV CLASS**   **NEXT CLASS** | **FRAMES**    **NO FRAMES**     **All Classes** |  |
| SUMMARY: NESTED \| FIELD \| CONSTR \| METHOD | DETAIL: FIELD \| CONSTR \| METHOD |  |


---

# *Copyright © 2007-2008*
